# Preparation of Self-Healing Antifogging Hard Coatings Using Carboxy-Functionalized Polysilsesquioxanes and Oligo(ethylene glycol)s

**DOI:** 10.3390/polym17182491

**Published:** 2025-09-15

**Authors:** Seiya Morinaga, Rione Baba, Chino Fujii, Yoshiro Kaneko

**Affiliations:** 1 Graduate School of Science and Engineering, Kagoshima University, 1-21-40 Korimoto, Kagoshima 890-0065, Japan; 2 Faculty of Engineering, Kagoshima University, 1-21-40 Korimoto, Kagoshima 890-0065, Japan

**Keywords:** antifogging coating, hard coating, self-healing, polysilsesquioxane, oligo(ethylene glycol)

## Abstract

Water-resistant antifogging hard coatings possessing self-healing properties were successfully prepared by applying *N*,*N*-dimethylformamide solutions containing the mixtures of carboxy-functionalized polysilsesquioxane (PSQ-2C) with oligo(ethylene glycol)s (OEGs; *n* = 2–6 and *n* = 2–4) at the feed functional group ratios (carboxy groups in PSQ-2C/hydroxy groups in OEG) of 10:1 and 4:1, respectively, onto oxygen plasma–treated glass substrates, followed by heat drying, water immersion, and room-temperature drying. The formation of ester bonds in the resulting coatings, indicating the presence of a cross-linked structure, was confirmed via Fourier-transform infrared/attenuated total reflectance spectroscopy. Notably, the coating prepared using PSQ-2C and tetraethylene glycol (OEG; *n* = 4) at a feed functional group ratio of 10:1 demonstrated no peeling or dissolution even after water immersion for 1 h, and its surface hardness, which was evaluated via the pencil scratch test, was 4H. Additionally, when exposed to water vapor generated from warm water at 40 °C at a distance of 2 cm, the coating maintained transparency for up to 85 s, confirming its excellent antifogging performance. Finally, the coating exhibited self-healing properties, as evidenced by the disappearance of scratches induced by a 5H pencil when the coating was left standing at 25 °C and 30% relative humidity for 5 min.

## 1. Introduction

Fogging occurs when condensed water vapor, in the form of small droplets, adheres to cool surfaces owing to the changes in environmental factors, such as humidity and temperature. Antifogging technologies are employed in various applications, ranging from commonplace items, such as goggles and food packaging, to solar panels and analytical/medical devices. A strategy for the prevention of fogging is to control the relative humidity (RH) and temperature by increasing the substrate surface temperature, creating a fog-free environment [1]. However, this method requires equipment and energy to maintain the elevated substrate surface temperature for preventing water droplet adhesion, leading to increased costs and other challenges. Therefore, the coating of the substrate surface to prevent fogging has emerged as a mainstream approach owing to its easy preparation, cost-effectiveness, and excellent antifogging properties. Numerous antifogging materials have been developed [2,3], and the application of these coatings can be generally classified into two main strategies.

The first strategy involves the use of superhydrophobic coatings, which reduce the surface energy of the substrate, consequently increasing the contact angle of water droplets and facilitating their easy removal via a slight incline. Hydrophobic antifogging coatings composed of hydrophobic molecules have been studied [4,5,6,7,8]. However, these hydrophobic molecules often exhibit weak adhesion to the substrate. Furthermore, the realization of superhydrophobicity requires the coating of surfaces with hydrophobic molecules and the roughening of the surface structure. The resulting fine surface structure can cause light scattering, consequently reducing the coating transparency. These challenges limit the widespread adoption of superhydrophobic coatings for antifogging applications.

The second strategy involves the improvement in surface wettability through coatings. It includes the use of hydrophilic coatings that form a thin, continuous water layer on the surface and water-absorbent coatings that absorb water droplets. These coatings are composed of inorganic materials, such as titanium dioxide and silica [9,10,11,12,13,14,15,16], or hydrophilic organic polymers. Titanium dioxide prevents fogging owing to its superhydrophilicity due to photocatalytic activity under ultraviolet (UV) light irradiation. However, the requirement of UV light limits its application in indoor or nighttime environments. Although silica-based coatings offer hydrophilic surfaces, their fabrication requires high-temperature processing, rendering them unsuitable for heat-sensitive polymer substrates and resulting in high energy consumption during coating production. Therefore, hydrophilic coatings based on inorganic materials have limited applications. Consequently, hydrophilic/water-absorbing antifogging coatings composed of readily available hydrophilic organic polymers have emerged as the prevalent choice for antifogging applications [17,18,19,20,21,22,23,24,25,26,27,28,29,30,31]. However, the inherent softness of these polymers renders their coatings susceptible to scratching and abrasion, which reduces their antifogging performance and transparency during applications. Therefore, organic–inorganic hybrid materials are gaining attention as antifogging hard coatings [32,33,34,35]. For example, silica nanoparticles were modified with 3-triethoxysilylpropyl methacrylate, followed by photopolymerization to prepare a hydrophobic cured coating. Thereafter, a hydrophilic organic polymer was applied to form a hydrophilic top layer, and the two coating layers were subsequently bonded [34].

Recently, hydrophilic silsesquioxanes (SQs), which are robust materials, have garnered attention for application in antifogging hard coatings. SQs are siloxane compounds comprising the repeating unit of RSiO_1.5_ (R = organic group or H) [36] and are obtained via the hydrolytic condensation of trifunctional organosilane compounds. SQs can form various structures, including the ladder-like, fully condensed cage (polyhedral oligomeric silsesquioxane (POSS)), incompletely condensed cage, and double-decker structures. A key advantage of SQs is that various organic groups can be introduced onto their side chains. Our research group has developed SQs with ammonium [37,38], carboxy [39], sulfo [40], and phosphonic acid groups [41] for ladder-like polySQs (PSQs) [42], and ammonium [43,44], carboxy [45,46], and imidazolium groups [47,48] for POSSs [49]. The diverse combinations of side chain substituents and structures enable the preparation of materials featuring a wide range of properties, rendering the resulting materials promising for various applications.

Antifogging hard coatings comprising random PSQs featuring amino [50] or glycidyl [51] side groups have been reported. Furthermore, our research group has developed POSS-based antifogging hard coatings. Specifically, we developed antifogging hard coatings based on polymers linked by amide bonds via the polycondensation of ammonium-functionalized POSS and carboxy-functionalized POSS (POSS-C) using condensation agents [52]. Although these coatings exhibited excellent antifogging and surface hardness, they were limited by delamination from the substrate upon water immersion. Therefore, our group developed an antifogging hard coating composed of POSS-C and oligo(ethylene glycol)s (OEGs, HO[CH_2_CH_2_O]*_n_*H, *n* = 1–6) [53]. The coating prepared using tetraethylene glycol (OEG; *n* = 4) exhibited excellent antifogging, surface hardness, and water resistance properties and maintained its coating state even after water immersion. However, in a water vapor exposure test, in which the coating was exposed to water vapor by placing it at a distance of 2 cm above warm water at 40 °C, the time required for fogging was ~10 s, indicating the need for further improvement in the antifogging performance. Furthermore, POSS-C used in these coatings requires a complex two-step reaction with low yields, posing a concern for practical applications.

Recently, increasing attention has been directed toward self-healing coatings. Currently, three main mechanisms of self-healing have been identified. The first mechanism involves intermolecular interactions, such as hydrogen bonding [54,55,56], metal–ligand coordination [57,58], and host–guest interactions [59,60], as well as dynamic and reversible covalent bonding, which can be realized through Diels–Alder (DA)/retro-DA reactions, imine bonds, and disulfide bonds, among others [61,62,63,64] for damage repair. The second mechanism comprises thermally triggered healing, in which heating promotes the motion of polymer chains, enabling them to re-entangle and restore the damaged region [65,66]. The third mechanism is based on viscoelastic recovery [67]. Self-healing coatings featuring the viscoelastic properties of polymers gradually recover from indentations over time.

Self-healing antifogging coatings based on the aforementioned mechanisms have been reported [68,69,70]. However, these coatings typically comprise hydrophilic polymers, resulting in low surface hardness. Although the incorporation of silica nanoparticles improves the hardness of the coatings to 4H in pencil scratch tests, water immersion is required to activate the self-healing behavior of these coatings [71]. Therefore, the development of self-healing antifogging hard coatings remains a considerable challenge.

Our research group had previously developed a soluble PSQ containing two carboxy groups in its repeating units (PSQ-2C) [45]. This PSQ formed a ladder-like structure owing to its low silanol content and high average molecular weight. In this study, we aimed to develop antifogging hard coatings featuring enhanced antifogging properties and self-healing capability using PSQ-2C, which was easy to prepare. The coating prepared by combining PSQ-2C with tetraethylene glycol at a feed functional group ratio (carboxy groups in PSQ-2C/hydroxy groups in OEG) of 10:1 exhibited a surface hardness of 4H in the pencil scratch test. Moreover, it demonstrated antifogging performance for 85 s in a water vapor exposure test conducted by placing it at a distance of 2 cm above warm water at 40 °C. Furthermore, the coating demonstrated excellent water resistance. Additionally, it exhibited self-healing properties, as evidenced by the disappearance of scratches induced by a 5H pencil on the coating after a standing period of ~5 min under ambient conditions (25 °C and 30% RH).

## 2. Materials and Methods

### 2.1. Materials

[3-(Triethoxysilyl)propyl]succinic anhydride (TESPSA; purity: ~93.0%), diethylene glycol (purity: ~99.5%), triethylene glycol (purity: ~99.0%), tetraethylene glycol (purity: ~95.0%), pentaethylene glycol (purity: ~95.0%), hexaethylene glycol (purity: ~98.0%), and tris(2,4-pentanedionato)chromium(III) (98%) were purchased from Tokyo Chemical Industry Co., Ltd. (Tokyo, Japan). Polyethylene glycol possessing an average molecular weight of 1000 (PEG1000) was purchased from Nacalai Tesque, Inc. (Kyoto, Japan). Ethanol (purity: ~99%) was purchased from Japan Alcohol Trading Co., Ltd. (Tokyo, Japan). *N*,*N*-dimethylformamide (DMF; purity: ~99.5%), hydrochloric acid (HCl; purity: 35–37%), diethyl ether (purity: ~99.5%), methanol (purity: ~99.5%), ethylene glycol (purity: ~99.5%), and methanol-*d*_4_ (CD_3_OD; purity: 99.8%) were purchased from FUJIFILM Wako Pure Chemical Co., Ltd. (Osaka, Japan). Tetrahydrofuran (THF; purity: 99.0%; stabilized with 2,6-di-*tert*-butyl-4-methylphenol) was purchased from Kanto Chemical Co., Inc. (Tokyo, Japan). All reagents and solvents were used without further purification.

### 2.2. Measurements

^1^H and ^29^Si nuclear magnetic resonance (NMR) spectra were recorded using an ECX-400 spectrometer (JEOL RESONANCE Inc., Tokyo, Japan). Number-average molecular weight (*M*_n_) and molecular weight distribution (*M*_w_/*M*_n_) were determined via gel permeation chromatography (GPC) using polystyrene standards. For GPC analysis, SHIMADZU CTO-20AC and SHIMADZU RID-20A systems (Shimadzu Corporation, Kyoto, Japan) were employed, and Shodex KF-803L (bead size: 6 μm; measurable molecular weight range: 1.0 × 10^2^–7.0 × 10^4^) and Shodex KF-805L (bead size: 6 μm; measurable molecular weight range: 1.0 × 10^2^–5.0 × 10^6^) columns (Resonac Corporation [formerly Showa Denko K.K.], Tokyo, Japan) were used. THF was used as the eluent and pumped through the system at a flow rate of 1.0 mL min^−1^ at 40 °C. The Fourier-transform infrared/attenuated total reflectance (FTIR/ATR) spectra were recorded using an IRSprit-T spectrometer (SHIMADZU CORPORATION, Kyoto, Japan). For evaluating their water resistance, the coatings were immersed in water at room temperature (~25 °C) for 1 h, after which water droplets were wiped off from the coating surface, and the state of the coatings was observed. Pencil hardness was measured using a pencil scratch tester (TP GIKEN Co., Osaka, Japan) at an angle of 45° under a load of 750 g. The pencils used were manufactured by Mitsubishi Pencil Co., Ltd. (Tokyo, Japan). The leads of the pencils were perpendicularly ground to create an angle of 90° before each pencil hardness measurement. The antifogging performance of the coatings was evaluated by placing the coating surface face down at a distance of 2 cm above warm water at 40 °C to expose the surface to water vapor (Figure S1). The water contact angles of the coatings were evaluated using a water-drop contact-angle meter (SImage Entry 6, Excimer, Inc., Kanagawa, Japan). The amount of water used for each droplet was 3.6 μL, and the contact angle of the droplet was measured using a charge-coupled device camera via the half-angle method. The self-healing performance was evaluated by placing the scratched coatings in a compact environmental test chamber (SH-242, ESPEC Corp., Osaka, Japan) at 25 °C and 30% RH. The healing behavior was visually observed after allowing the coating to stand for 5 min under the aforementioned conditions. Differential scanning calorimetry (DSC) analyses were performed on a DSC-60 Plus instrument (SHIMADZU Co., Kyoto, Japan). The sample was placed in an aluminum capsule and cooled to −100 °C at a rate of 20 °C min^−1^ under a nitrogen flow (100 mL min^−1^) and subsequently heated from −100 °C to 100 °C at the same rate. The data from the curve in the third set (from −100 °C to 100 °C at a rate of 20 °C min^−1^) was used to eliminate the heat histories in the samples.

### 2.3. Preparation of PSQ-2C

PSQ-2C was prepared using a previously reported procedure with minor modifications (Scheme S1) [45]. A 0.1 mol L^−1^ aqueous solution of HCl (125 mL; 12.5 mmol) was added to TESPSA (1.602 g; 5.0 mmol) under stirring at 80 °C for 1 h. The solution was subsequently heated at ~50 °C in an open system until the solvent was completely evaporated. The resulting crude product was further heated in an oven at 100 °C for 2 h. After cooling to room temperature, methanol (30 mL) was added to the product. The resulting solution was concentrated to ~10 mL using a rotary evaporator and reprecipitated from diethyl ether (300 mL). The diethyl ether–insoluble portion was isolated via decantation and washed with diethyl ether (~20 mL; three times). After drying the product under reduced pressure, it was dissolved in water (20 mL). The aqueous solution was heated at ~50 °C in an open system until the solvent was completely evaporated. The resulting solid product was subsequently heated in an oven at 100 °C for 2 h and crushed using a mortar, yielding powdered PSQ-2C (0.932 g; yield: 88%, calculated based on the ideal chemical formula of the repeating unit of PSQ-2C [SiO_1.5_(CH_2_)_3_CH(COOH)CH_2_COOH; FW = 211.24]). ^1^H NMR (400 MHz, CD_3_OD; Figure S2): δ 2.85–2.55 (3H, br, –C*H*COOH and –C*H*_2_COOH), and δ 1.74–1.53 (4H, br, –SiCH_2_C*H*_2_C*H*_2_CH–), and δ 0.70 (2H, br, –SiC*H*_2_CH_2_CH_2_CH–). ^29^Si NMR (79.4 MHz; CD_3_OD): δ −65.4–−69.7 (T^3^).

### 2.4. Ethyl Esterification of the Carboxy Groups in PSQ-2C

To calculate the average molecular weight of PSQ-2C using GPC, the carboxy groups were ethyl esterified. The ethyl esterification of PSQ-2C was performed as follows (Scheme S2). Ethanol (17.5 mL) was added to PSQ-2C (0.106 g; 0.5 mmol unit), followed by the addition of concentrated HCl (~125 μL; 1.5 mmol). Subsequently, the mixture was refluxed at 90 °C for 3 h. Afterward, the mixture was heated in an open system at ~50 °C until ethanol was completely evaporated. This process was repeated two times to obtain a solid product, yielding ethyl-esterified PSQ-2C (0.072 g; yield: 54%, calculated based on the ideal chemical formula of the repeating unit of PSQ-2C [SiO_1.5_(CH_2_)_3_CH(COOCH_2_CH_3_)CH_2_COOCH_2_CH_3_; FW = 267.35]). ^1^H NMR (400 MHz; CD_3_OD; Figure S3): δ 4.11 (4H, br, –COOC*H*_2_CH_3_), δ 2.84–2.53 (3H, br, –C*H*COOCH_2_CH_3_ and –C*H*_2_COOCH_2_CH_3_), δ 1.73–1.50 (4H, br, –SiCH_2_C*H*_2_C*H*_2_CH–), δ 1.24 (6H, br, –COOCH_2_C*H*_3_), and δ 0.69 (2H, br, –SiC*H*_2_CH_2_CH_2_CH–). The *M*_n_ of the resulting ethyl-esterified PSQ-2C was estimated as 2.88 × 10^4^, and the *M*_w_/*M*_n_ value was 2.98 (Figure S4).

### 2.5. Preparation of Coatings

A glass substrate (48 × 28 mm; thickness = 1.3 mm) was ultrasonically cleaned in ethanol for ~2 min and treated to render the surface hydrophilic using plasma equipment (Plasma Modifier PM100, Yamato Scientific Co., Ltd., Tokyo, Japan). Plasma treatment was performed by flowing oxygen at a rate of 100 mL min^−1^ for 30 s, followed by plasma irradiation for 3 min. To ensure a consistent coating area, a Teflon seal was affixed to the glass substrate, creating an area of 700 mm^2^ (25 × 28 mm). A representative example of the reaction combining PSQ-2C and tetraethylene glycol at a feed functional group ratio (carboxy (COOH) group in PSQ-2C/hydroxy (OH) group in tetraethylene glycol) of 10:1 is described as follows. PSQ-2C (0.0422 g; 0.2 mmol based on carboxy groups) and tetraethylene glycol (0.0041 g; 0.02 mmol based on hydroxy groups) were dissolved in DMF (0.2 mL). The mixture was heated and stirred at 80 °C for 1 h, yielding a homogeneous solution. This solution was applied onto the aforementioned oxygen plasma–treated glass substrate (700 mm^2^; 25 × 28 mm). The coated substrate was heated in an open system on a hot plate at a set temperature of 50 °C for 2 h to remove the solvent (DMF). Thereafter, it was heated in an oven at 150 °C for 1 h, immersed in water at room temperature for 1 h, and subsequently dried by standing at room temperature to yield the PSQ-2C/OEG (*n* = 4; COOH/OH = 10:1) coating. Here, COOH/OH represents the feed functional group ratio of carboxy groups in PSQ-2C to hydroxy groups in OEG, and *n* indicates the degree of polymerization of OEGs. Other PSQ-2C/OEG coatings were prepared in a similar manner.

## 3. Results and Discussion

### 3.1. Preparation of PSQ-2C/OEG Coatings

**Scheme 1:**
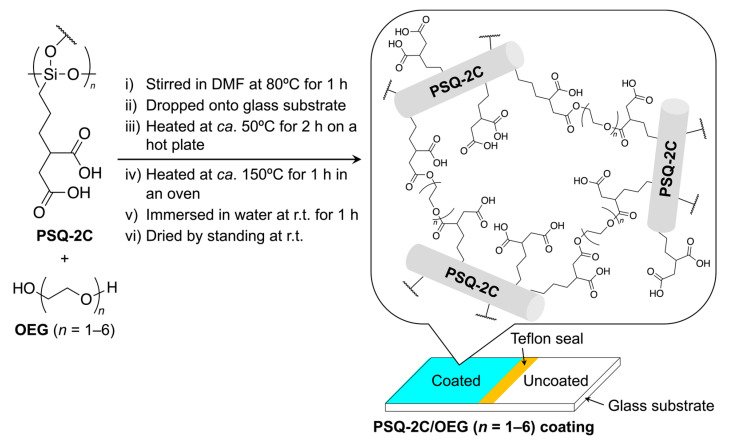
Preparation of PSQ-2C/OEG (*n* = 1–6) Coatings.

The PSQ-2C/OEG coatings were prepared as follows. First, a DMF solution of PSQ-2C and OEG was heated at 80 °C under stirring. Thereafter, the resulting solution was applied to an oxygen plasma–treated glass substrate. The coated substrate was subsequently heated for 2 h at ~50 °C in an open system to remove DMF. Thereafter, it was further heated in an oven at 150 °C for 1 h to promote esterification between PSQ-2C and OEG (Scheme 1i–iv). However, after heating, dehydration condensation occurred between the carboxy groups in the same repeating unit, and the formation of a succinic anhydride structure (exhibiting an absorption peak at approximately 1780 cm^−1^) was confirmed via FTIR/ATR analysis (Figures S5–S7). Therefore, the as-formed succinic anhydride structure was subsequently hydrolyzed to regenerate the carboxy groups by immersing the coating in water. Afterward, the coating was dried by standing it at room temperature, yielding the PSQ-2C/OEG coatings (Scheme 1v,vi). The thickness of all coatings was approximately 30–40 µm. The resulting coatings were visually transparent. Moreover, as a representative example, a coating (approximately 30 μm thick) composed of PSQ-2C and tetraethylene glycol (COOH/OH = 10:1) exhibited over 96% transmittance in the visible wavelength region, as confirmed by the UV–Vis spectrum measured with a glass substrate as the background (Figure S8).

### 3.2. Water Resistance of PSQ-2C/OEG Coatings

**Figure 1:**
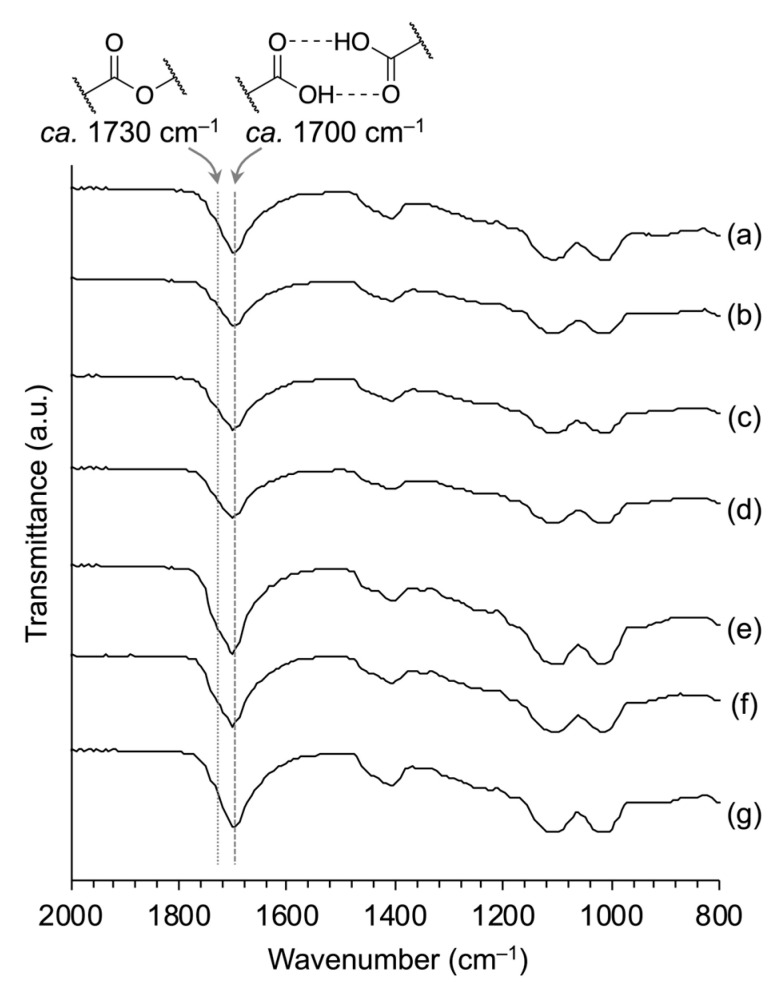
FTIR/ATR spectra of the PSQ-2C/OEG ((a) *n* = 1, (b) *n* = 2, (c) *n* = 3, (d) *n* = 4, (e) *n* = 5, and (f) *n* = 6) coatings featuring a COOH/OH feed functional group ratio of 10:1 after water immersion for 1 h and (g) PSQ-2C coating.

**Figure 2:**
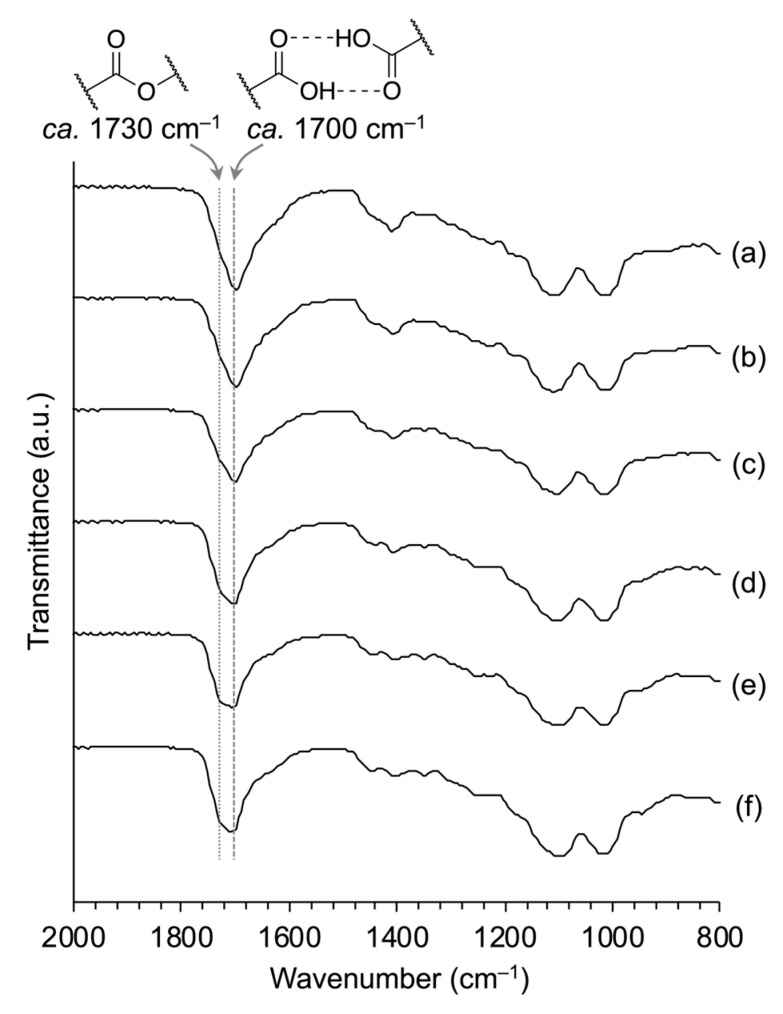
FTIR/ATR spectra of the PSQ-2C/OEG ((a) *n* = 1, (b) *n* = 2, (c) *n* = 3, (d) *n* = 4, (e) *n* = 5, and (f) *n* = 6) coatings featuring a COOH/OH feed functional group ratio of 4:1 after water immersion for 1 h.

The PSQ-2C/OEG (*n* = 2–6; COOH/OH = 10:1) and PSQ-2C/OEG (*n* = 2–4; COOH/OH = 4:1) coatings exhibited excellent water resistance, as they did not peel off or dissolve even after being immersed in water for 1 h (runs 4, 5, 7, 8, 10, 11, 13, and 16 in Table 1). The FTIR/ATR spectra of these coatings demonstrated absorption peaks attributed to the C=O bonds of ester groups (Figure 1b–f and Figure 2b–d), suggesting the progression of cross-linking reactions. In the case of the COOH/OH feed functional group ratio of 10:1, the lower OEG content resulted in the involvement of fewer carboxy groups in ester bond formation within the PSQ-2C component, leading to a larger number of unreacted carboxy groups. These unreacted carboxy groups plausibly contributed to the strong adhesion of the coating to the glass substrate surface, presumably via hydrogen bonding with silanol groups. Even at a COOH/OH feed functional group ratio of 4:1, when *n* was small (*n* = 2–4), the weight ratio of PSQ-2C in the overall coating was high. Here, similar to the case of the coating comprising a COOH/OH feed functional group ratio of 10:1, the carboxy groups in the PSQ-2C component probably contribute to the strong adhesion of the coating to the glass substrate.

**Figure 3:**
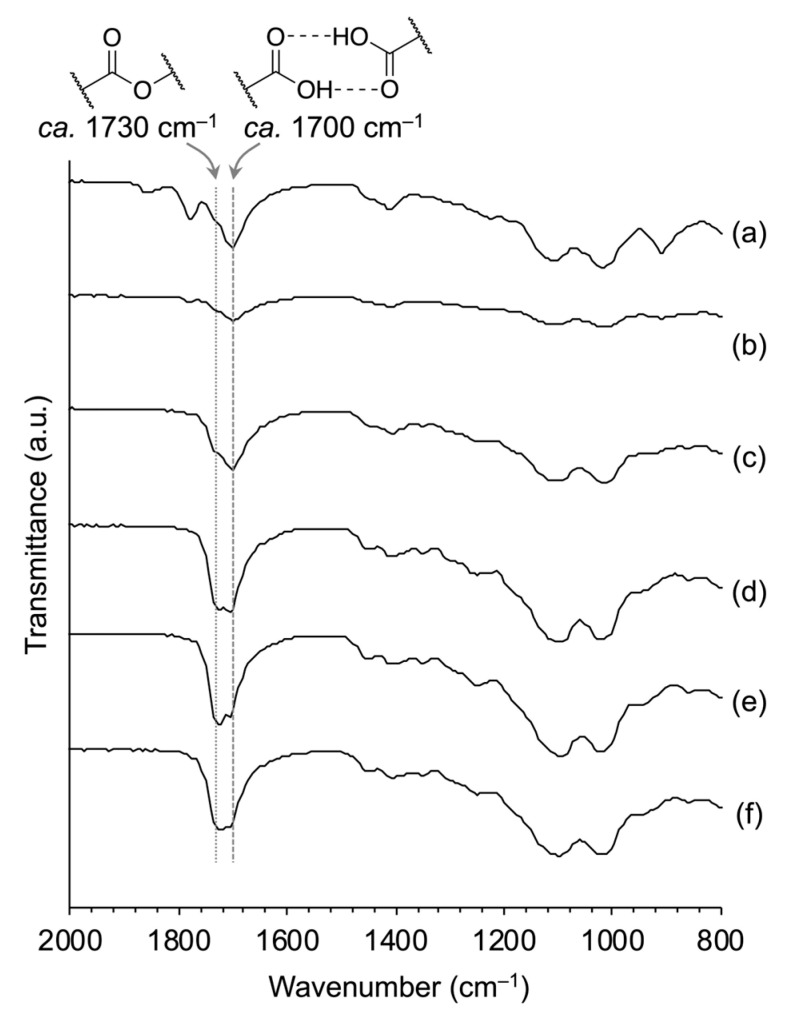
FTIR/ATR spectra of the PSQ-2C/OEG ((a) *n* = 1, (b) *n* = 2, (c) *n* = 3, (d) *n* = 4, (e) *n* = 5, and (f) *n* = 6) coatings featuring a COOH/OH feed functional group ratio of 2:1 after water immersion for 1 h.

Conversely, the PSQ-2C/OEG coatings featuring the COOH/OH feed functional group ratios of 4:1 (*n* = 5 and 6) and 2:1 (*n* = 1–6) peeled off from the glass substrate during water immersion (runs 3, 6, 9, 12, 14, 15, 17, and 18 in Table 1). Because the coatings did not dissolve in water, the cross-linking reactions between PSQ-2C and OEG are considered to have sufficiently progressed. Furthermore, the FTIR/ATR spectra confirmed the formation of ester bonds (Figure 2e,f and Figure 3b–f). However, compared with the coatings comprising a COOH/OH feed functional group ratio of 10:1, the higher OEG content decreased the number of unreacted carboxy groups within the PSQ-2C component that were not involved in the ester bond formation. Consequently, the interaction between the carboxy groups in the PSQ-2C component and the silanol groups on the glass substrate surface, plausibly via hydrogen bonding, was insufficient. Water was presumed to have infiltrated the interface during immersion, causing the coatings to peel off.

Additionally, the PSQ-2C/ethylene glycol (OEG; *n* = 1; COOH/OH = 10:1 and 4:1) coatings occasionally exhibited a sticky surface (runs 1 and 2 in Table 1). This is assumed to be due to the evaporation of ethylene glycol during heat treatment before the formation of ester bonds between the carboxy groups of PSQ-2C and the hydroxy groups of ethylene glycol, resulting in coatings solely composed of PSQ-2C. Furthermore, the FTIR/ATR spectra demonstrated no absorption peaks attributed to the C=O bonds of ester groups (Figure 1a and Figure 2a). Although PSQ-2C is water-soluble, it does not easily dissolve in water at room temperature. Therefore, the coatings remained intact after water immersion. However, the coating surfaces were noticeably sticky after their removal from water, rendering them unsuitable for application as antifogging coatings.

### 3.3. Surface Hardness of PSQ-2C/OEG Coatings

The surface hardness of the PSQ-2C/OEG coatings, including the PSQ-2C/OEG (*n* = 2–6; COOH/OH = 10:1) and PSQ-2C/OEG (*n* = 2–4; COOH/OH = 4:1) coatings, which neither peeled off nor dissolved and did not exhibit stickiness during the aforementioned water-resistance tests, was evaluated using a pencil scratch tester. Notably, the PSQ-2C/OEG (*n* = 4–6; COOH/OH = 10:1) and PSQ-2C/OEG (*n* = 3 and 4; COOH/OH = 4:1) coatings exhibited relatively high surface hardness, reaching 4H (runs 8, 10, 11, 13, and 16 in Table 1). This increase in hardness is attributed to the rigidity of the inorganic framework of PSQ-2C and to the formation of a cross-linked structure.

Comparing with the surface hardness of 6H determined for the POSS-C/OEG (*n* = 4; COOH/OH = 2:1) coating in our previous study [53], the hardness of the current coatings decreased. This reduction is plausibly because of the larger amounts of organic components present in the side chains of PSQ-2C than those of POSS-C. However, the surface hardness (4H) of the PSQ-2C/OEG coatings developed in this study is higher than that (approximately 2H) of acrylic resin and human nails.

### 3.4. Antifogging Property of PSQ-2C/OEG Coatings

**Figure 4:**
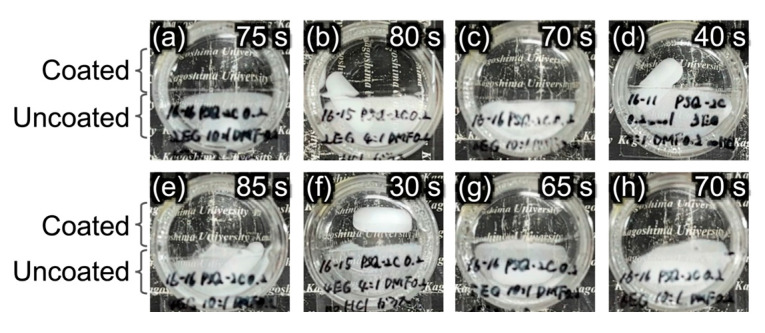
Antifogging behavior of the PSQ-2C/OEG ((**a**) *n* = 2; COOH/OH = 10:1, (**b**) *n* = 2; COOH/OH = 4:1, (**c**) *n* = 3; COOH/OH = 10:1, (**d**) *n* = 3; COOH/OH = 4:1, (**e**) *n* = 4; COOH/OH = 10:1, (**f**) *n* = 4; COOH/OH = 4:1, (**g**) *n* = 5; COOH/OH = 10:1, and (**h**) *n* = 6; COOH/OH = 10:1) coatings upon exposure to water vapor.

To evaluate the antifogging performance of the water-resistant PSQ-2C/OEG (*n* = 2–6; COOH/OH = 10:1) and PSQ-2C/OEG (*n* = 2–4; COOH/OH = 4:1) coatings, the coated surfaces were placed face down at a distance of 2 cm above warm water at 40 °C to expose them to water vapor, and their fogging behavior was subsequently observed. The antifogging behavior of each coating is illustrated in Figure 4, and the time taken for the initiation of fogging is summarized in Table 1.

The PSQ-2C/OEG coatings comprising a low feed ratio of OEG cross-linkers (COOH/OH = 10:1) required a longer time for fogging than the coatings featuring a COOH/OH feed functional group ratio of 4:1, demonstrating superior antifogging properties. Particularly, the PSQ-2C/OEG (*n* = 4; COOH/OH = 10:1) coating demonstrated water resistance and high surface hardness (4H) and maintained a fog-free state for up to 85 s upon exposure to water vapor, exhibiting an excellent antifogging performance (Figure 4e and run 10 in Table 1).

Several factors contribute to this superior antifogging performance of the PSQ-2C/OEG (*n* = 4; COOH/OH = 10:1) coating compared to the other analyzed coatings. First, the lower feed ratio of OEG resulted in a higher content of unreacted carboxy groups in the PSQ-2C components, which enhanced the affinity of the coatings toward water, rendering them more effective as hydrophilic coatings. Additionally, the lower feed ratio of OEG led to a lower cross-linking density, facilitating the formation of pores within the coatings. These pores enabled the coatings to absorb a large amount of water, contributing to the water-absorbing capability of these coatings. In fact, the water absorption ratio—calculated as the weight of absorbed water relative to the coating weight—after 3 min of exposure to water vapor was approximately 9.7 wt% for the 10:1 coating and approximately 6.8 wt% for the 4:1 coating, indicating that the 10:1 coating absorbed more water. A detailed discussion on the pores is provided in the subsequent section on water contact angle measurements.

### 3.5. Water Contact Angles of PSQ-2C/OEG Coatings

**Figure 5:**
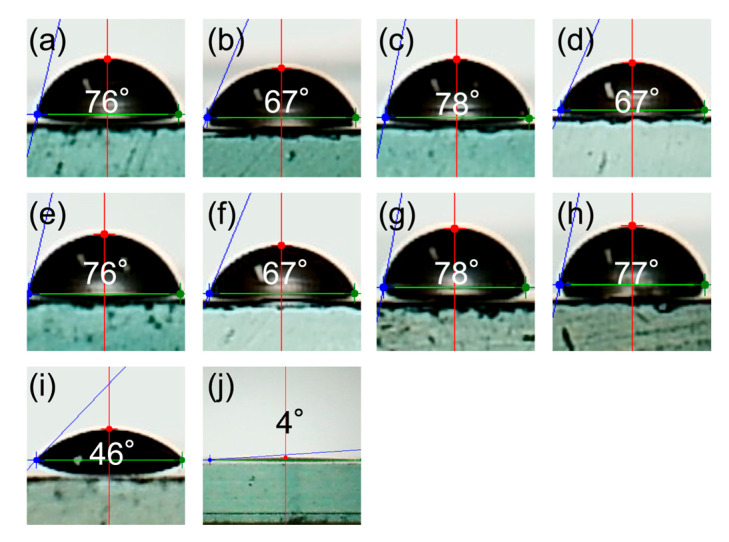
Water contact angles of the PSQ-2C/OEG ((**a**) *n* = 2; COOH/OH = 10:1, (**b**) *n* = 2; COOH/OH = 4:1, (**c**) *n* = 3; COOH/OH = 10:1, (**d**) *n* = 3; COOH/OH = 4:1, (**e**) *n* = 4; COOH/OH = 10:1, (**f**) *n* = 4; COOH/OH = 4:1, (**g**) *n* = 5; COOH/OH = 10:1, and (**h**) *n* = 6; COOH/OH = 10:1), (**i**) PSQ-2C, and (**j**) PEG1000 coatings.

To investigate the antifogging mechanism of the PSQ-2C/OEG coatings, their water contact angles were measured (Figure 5). First, the water contact angles of the raw material PSQ-2C and PEG1000, which is a substitute compound for OEGs, were measured. Because OEGs (*n* = 1–6) are liquids, PEG1000, which is a solid compound with the same repeating unit structure, is chosen as the substitute. The water contact angles of the PSQ-2C and PEG1000 coatings were 46° and 4°, respectively (runs 19 and 20 in Table 1 and Figure 5i,j). However, the water contact angles of all the PSQ-2C/OEG (*n* = 2–6; COOH/OH = 10:1 and 4:1) coatings were >67° (runs 4, 5, 7, 8, 10, 11, 13, and 16 in Table 1 and Figure 5a–h), demonstrating higher values than those of the raw material PSQ-2C and PEG1000 substitute.

This increase in the water contact angle of the PSQ-2C/OEG (*n* = 2–6; COOH/OH = 10:1 and 4:1) coatings is plausibly due to the network structure formed by the cross-linking reaction via ester bonds in the PSQ-2C/OEG coatings, creating pores within the coatings. The larger water contact angles of the PSQ-2C/OEG coatings than those of the raw materials can be attributed to the Lotus effect. When the coatings are exposed to water vapor, water molecules penetrate the pores of the coating in the form of vapor, and upon cooling, they form a uniform water layer, imparting excellent antifogging properties to the coatings. Considering that the water contact angles of the PSQ-2C/OEG coatings are not extremely small, their antifogging performance can be attributed not only to their hydrophilicity but also primarily to their water-absorbing properties.

### 3.6. Self-Healing Property of PSQ-2C/OEG Coatings

**Figure 6:**
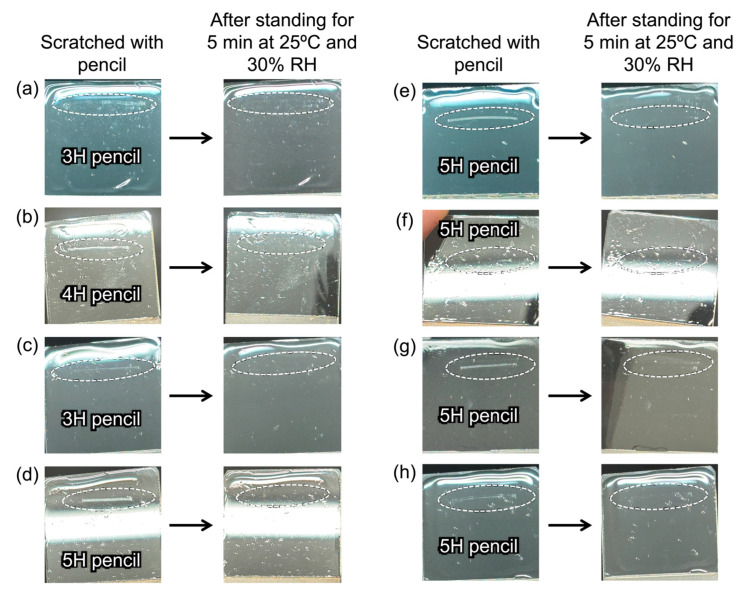
Appearances after scratching the PSQ-2C/OEG ((**a**) *n* = 2; COOH/OH = 10:1, (**b**) *n* = 2; COOH/OH = 4:1, (**c**) *n* = 3; COOH/OH = 10:1, (**d**) *n* = 3; COOH/OH = 4:1, (**e**) *n* = 4; COOH/OH = 10:1, (**f**) *n* = 4; COOH/OH = 4:1, (**g**) *n* = 5; COOH/OH = 10:1, and (**h**) *n* = 6; COOH/OH = 10:1) coatings using a pencil scratch tester equipped with a pencil featuring a hardness value one rank higher than the hardness values listed in Table 1 and standing the samples at 25 °C and 30% RH.

In the aforementioned pencil scratch test, no scratches were induced by the pencils featuring hardness values equal to or lower than those listed in Table 1, whereas clear scratches were induced by the pencils that were one rank harder. However, these scratches naturally disappeared without any specific treatment, suggesting a self-healing behavior of the PSQ-2C/OEG coatings. Therefore, the self-healing property of the PSQ-2C/OEG coatings was investigated by placing the scratched samples in a testing chamber with controlled temperature and humidity. Using a pencil scratch tester equipped with a pencil featuring a hardness value one rank higher than the hardness values listed in Table 1, scratches were induced on the PSQ-2C/OEG coatings, and the samples were subsequently held at 25 °C and 30% RH for observation. All the coatings demonstrated self-healing behavior, with the scratches disappearing within 5 min (Figure 6). Particularly, the PSQ-2C/OEG (*n* = 4; COOH/OH = 10:1) coating exhibited multiple exceptional properties—water resistance, a surface hardness of 4H, and an antifogging time of 85 s (run 10 in Table 1). Furthermore, scratches induced by a 5H pencil on the coating completely healed within 5 min (Figure 6e).

**Figure 7:**
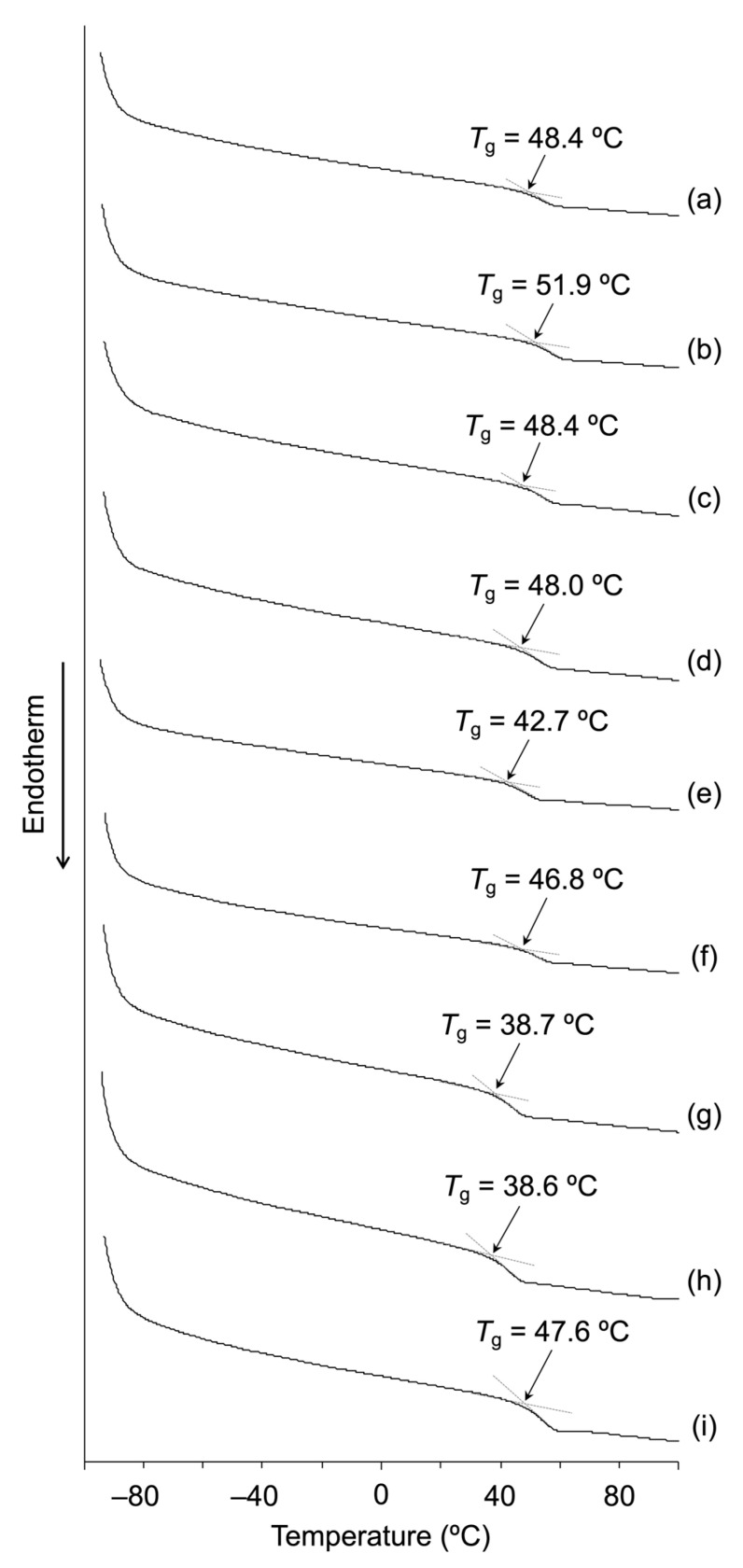
DSC curves of the PSQ-2C/OEG ((a) *n* = 2; COOH/OH = 10:1, (b) *n* = 2; COOH/OH = 4:1, (c) *n* = 3; COOH/OH = 10:1, (d) *n* = 3; COOH/OH = 4:1, (e) *n* = 4; COOH/OH = 10:1, (f) *n* = 4; COOH/OH = 4:1, (g) *n* = 5; COOH/OH = 10:1, and (h) *n* = 6; COOH/OH = 10:1) coatings and (i) PSQ-2C coating.

The self-healing mechanism of the PSQ-2C/OEG coatings was investigated. Generally, the self-healing mechanisms of materials include the following: (i) damage repair through physical interactions or dynamic/reversible covalent bonding at the interface, (ii) heating of the coating to induce the motion of polymer chains, leading to material softening and re-entanglement for healing, and (iii) viscoelastic recovery, in which deformations are gradually restored over time because of the viscoelastic properties of the material. If the self-healing behavior of the PSQ-2C/OEG coatings were due to mechanism (i), namely, physical interactions or dynamic/reversible covalent bonding at the interface, deep scratches induced by a cutter should have been repaired. However, the scratches induced by a cutter did not undergo self-healing (Figure S9). Based on this result, the self-healing of the PSQ-2C/OEG coatings is not likely to originate from mechanism (i). Furthermore, DSC measurements performed on the coatings partially peeled off from the glass substrate revealed that the glass transition temperature (*T*_g_) ranged from 43 °C to 52 °C (Figure 7). Furthermore, because the PSQ-2C/OEG coatings demonstrated self-healing behavior even under ambient conditions (25 °C), mechanism (ii), which involves material softening through heating, is considered implausible.

Based on the aforementioned results, the self-healing property of the PSQ-2C/OEG coatings against scratches induced by a 5H pencil is attributed to their viscoelasticity (mechanism (iii)). The rigidity of PSQ-2C enables a hard coating surface, rendering the formation of deep scratches that tear the surface difficult, even when scratched using a 5H pencil, which causes only temporary physical indentations. Furthermore, the flexibility of OEG contributes to the gradual recovery of these indentations through viscoelasticity, ultimately leading to the self-healing of scratches. Although scratches induced by a cutter are irreversible and cannot be healed, representing a current limitation, these findings emphasize the potential of PSQ-2C/OEG systems for practical antifogging coatings requiring mechanical robustness and self-healing capability.

## 4. Conclusions

In this study, water-resistant antifogging hard coatings possessing self-healing properties were successfully prepared by applying DMF solutions containing the mixtures of PSQ-2C with OEGs (*n* = 2–6 and *n* = 2–4) at the COOH/OH feed functional group ratios of 10:1 and 4:1, respectively, onto oxygen plasma–treated glass substrates, followed by heat drying, water immersion, and room-temperature drying. Notably, the coating prepared using PSQ-2C and tetraethylene glycol (OEG; *n* = 4) at a feed functional group ratio of 10:1 exhibited no peeling or dissolution even after 1 h of water immersion, and its surface hardness, which was evaluated via the pencil scratch test, was 4H. Furthermore, in the water vapor exposure test (in which the coating was exposed to water vapor by placing it at a distance of 2 cm above warm water at 40 °C), the coating maintained transparency for up to 85 s, demonstrating an excellent antifogging performance. Another exceptional feature of the prepared coating was its self-healing ability against scratches. The scratches induced by a 5H pencil disappeared after standing the coating for 5 min in a chamber at 25 °C and 30% RH. In comparison with our previously reported POSS-C/OEG coatings [53], which achieved a higher surface hardness (6H) but maintained transparency for only 10 s under identical antifogging conditions, the present PSQ-2C/OEG system shows a substantially improved antifogging performance (85 s) while also offering the added value of self-healing capability. The as-developed antifogging coating successfully combines high surface hardness (4H) with a unique self-healing property, enabling recovery even from the scratches caused by harder materials.

In the future, considering the continuous shift toward lighter vehicle bodies, the use of resin materials for automotive windows is expected to increase, leading to a higher demand for antifogging hard coatings applicable to resin substrates. The development of antifogging hard coatings suitable for resin surfaces is currently underway.

## Figures and Tables

**Table 1:** Summary of water resistance, surface hardness, antifogging performance, and water contact angle of PSQ-2C/OEG coatings.

Run	Coating	Feed Functional Group Ratio COOH/OH ^a^	Water Resistance ^b^	Surface Hardness ^c^	Time to Keep Antifogging ^d^ (s)	Water Contact Angle ^e^
1	PSQ-2C/OEG (*n* = 1)	10:1	sticky	–	–	–
2	PSQ-2C/OEG (*n* = 1)	4:1	sticky	–	–	–
3	PSQ-2C/OEG (*n* = 1)	2:1	peeled off	–	–	–
4	PSQ-2C/OEG (*n* = 2)	10:1	not peeled off, not dissolved	2H	75	76°
5	PSQ-2C/OEG (*n* = 2)	4:1	not peeled off, not dissolved	3H	80	67°
6	PSQ-2C/OEG (*n* = 2)	2:1	peeled off	–	–	–
7	PSQ-2C/OEG (*n* = 3)	10:1	not peeled off, not dissolved	2H	70	78°
8	PSQ-2C/OEG (*n* = 3)	4:1	not peeled off, not dissolved	4H	40	67°
9	PSQ-2C/OEG (*n* = 3)	2:1	peeled off	–	–	–
10	PSQ-2C/OEG (*n* = 4)	10:1	not peeled off, not dissolved	4H	85	76°
11	PSQ-2C/OEG (*n* = 4)	4:1	not peeled off, not dissolved	4H	30	67°
12	PSQ-2C/OEG (*n* = 4)	2:1	peeled off	–	–	–
13	PSQ-2C/OEG (*n* = 5)	10:1	not peeled off, not dissolved	4H	65	78°
14	PSQ-2C/OEG (*n* = 5)	4:1	peeled off	–	–	–
15	PSQ-2C/OEG (*n* = 5)	2:1	peeled off	–	–	–
16	PSQ-2C/OEG (*n* = 6)	10:1	not peeled off, not dissolved	4H	70	77°
17	PSQ-2C/OEG (*n* = 6)	4:1	peeled off	–	–	–
18	PSQ-2C/OEG (*n* = 6)	2:1	peeled off	–	–	–
19	PSQ-2C	–	dissolved	3H	>120	46°
20	PEG1000	–	dissolved	<2B	>120	4°

^a^ Feed functional group ratio of the COOH group in PSQ-2C to the OH group in OEG. ^b^ Coated glass substrate was immersed in water at room temperature for 1 h and subsequently taken out to observe the appearance of the coating. ^c^ Surface hardness of the coatings was evaluated using a pencil scratch tester. ^d^ Antifogging properties were determined by placing the coated glass substrate, with the coated surface facing downward, at a distance of 2 cm above warm water at 40 °C to expose it to water vapor and observing the fogging behavior. ^e^ Water contact angle was measured 10 s after dropping a water droplet.

## Data Availability

All data supporting the findings of this study are available within the article and its Supplementary Materials.

## Supplementary Materials

The following supporting information can be downloaded at: https://www.mdpi.com/article/10.3390/polym17182491/s1. Preparation (Scheme S1) and ethyl esterification (Scheme S2) of PSQ-2C; photograph of the equipment used for antifogging evaluation (Figure S1); ^1^H NMR spectra of PSQ-2C (Figure S2) and ethyl-esterified PSQ-2C (Figure S3); GPC curve of ethyl-esterified PSQ-2C (Figure S4); FTIR/ATR spectra of the PSQ-2C/OEG (*n* = 1–6) coatings before water immersing (Figures S5–S7); UV–Vis spectrum of the PSQ-2C/OEG (*n* = 4; COOH/OH = 10:1) coating (Figure S8); appearance of PSQ-2C/OEG (*n* = 4, COOH/OH = 10:1) after being scratched with a cutter (Figure S9).

## Abbreviations

The following abbreviations are used in this manuscript:

SQ	Silsesquioxane
PSQ	Polysilsesquioxane
POSS	Polyhedral oligomeric silsesquioxane
POSS-C	Carboxy-functionalized polyhedral oligomeric silsesquioxane
TESPSA	[3-(Triethoxysilyl)propyl]succinic anhydride
PSQ-2C	Carboxy-functionalized polysilsesquioxane
OEG	Oligo(ethylene glycol)
DMF	*N*,*N*-Dimethylformamide
DMSO	Dimethyl sulfoxide
THF	Tetrahydrofuran
PEG1000	Polyethylene glycol with an average molecular weight of 1000
FTIR/ATR	Fourier-transform infrared spectroscopy/attenuated total reflectance
GPC	Gel permeation chromatography
DSC	Differential scanning calorimetry

## References

[1] Durán I.R., Laroche G. (2019). Current trends, challenges, and perspectives of anti-fogging technology: Surface and material design, fabrication strategies, and beyond. Prog. Mater. Sci..

[2] Wang Y., Gong X. (2017). Special oleophobic and hydrophilic surfaces: Approaches, mechanisms, and applications. J. Mater. Chem. A.

[3] Zhao J., Song L., Ming W. (2018). Antifogging and frost-resisting polymeric surfaces. Adv. Polym. Sci..

[4] Chen Y., Zhang Y., Shi L., Li J., Xin Y., Yang T., Guo Z. (2012). Transparent superhydrophobic/superhydrophilic coatings for self-cleaning and anti-fogging. Appl. Phys. Lett..

[5] Sun Z., Liao T., Liu K., Jiang L., Kim J.H., Dou S.X. (2014). Fly-eye inspired superhydrophobic anti-fogging inorganic nanostructures. Small.

[6] Shang Q., Zhou Y. (2016). Fabrication of transparent superhydrophobic porous silica coating for self-cleaning and anti-fogging. Ceram. Int..

[7] Syafiq A., Vengadaesvaran B., Ahmed U., Rahim N.A., Pandey A.K., Bushroa A.R., Ramesh K., Ramesh S. (2020). Facile synthesize of transparent hydrophobic nano-CaCO_3_ based coatings for self-cleaning and anti-fogging. Mater. Chem. Phys..

[8] Qiu Z., Lin H., Zeng L., Liang Y., Zeng C., Hong R. (2022). Ultra-scratch-resistant, hydrophobic and transparent organosilicon-epoxy-resin coating with a double cross-link structure. Appl. Sci..

[9] Watanabe T., Nakajima A., Wang R., Minabe M., Koizumi S., Fujishima A., Hashimoto K. (1999). Photocatalytic activity and photoinduced hydrophilicity of titanium dioxide coated glass. Thin Solid Films.

[10] Fujishima A., Rao T.N., Tryk D.A. (2000). Titanium dioxide photocatalysis. J. Photochem. Photobiol. C Photochem. Rev..

[11] Tricoli A., Righettoni M., Pratsinis S.E. (2009). Anti-fogging nanofibrous SiO_2_ and nanostructured SiO_2_-TiO_2_ films made by rapid flame deposition and in situ annealing. Langmuir.

[12] Lai Y., Tang Y., Gong J., Gong D., Chi L., Lin C., Chen Z. (2012). Transparent superhydrophobic/superhydrophilic TiO_2_-based coatings for self-cleaning and anti-fogging. J. Mater. Chem..

[13] Park J.T., Kim J.H., Lee D. (2014). Excellent anti-fogging dye-sensitized solar cells based on superhydrophilic nanoparticle coatings. Nanoscale.

[14] Lai Y., Huang J., Cui Z., Ge M., Zhang K.Q., Chen Z., Chi L. (2016). Recent advances in TiO_2_-based nanostructured surfaces with controllable wettability and adhesion. Small.

[15] Chemin J.B., Bulou S., Baba K., Fontaine C., Sindzingre T., Boscher N.D., Choquet P. (2018). Transparent anti-fogging and self-cleaning TiO_2_/SiO_2_ thin films on polymer substrates using atmospheric plasma. Sci. Rep..

[16] Xi R., Wang Y., Wang X., Lv J., Li X., Li T., Zhang X., Du X. (2020). Ultrafine nano-TiO_2_ loaded on dendritic porous silica nanoparticles for robust transparent antifogging self-cleaning nanocoatings. Ceram. Int..

[17] Howarter J.A., Youngblood J.P. (2008). Self-cleaning and next generation anti-fog surfaces and coatings. Macromol. Rapid. Commun..

[18] Tahk D., Kim T.I., Yoon H., Choi M., Shin K., Suh K.Y. (2010). Fabrication of antireflection and antifogging polymer sheet by partial photopolymerization and dry etching. Langmuir.

[19] Chevallier P., Turgeon S., Sarra-Bournet C., Turcotte R., Laroche G. (2011). Characterization of multilayer anti-fog coatings. ACS Appl. Mater. Interfaces.

[20] Lee H., Alcaraz M.L., Rubner M.F., Cohen R.E. (2013). Zwitter-wettability and antifogging coatings with frost-resisting capabilities. ACS Nano.

[21] Zhao J., Meyer A., Ma L., Ming W. (2013). Acrylic coatings with surprising antifogging and frost-resisting properties. Chem. Commun..

[22] Zhao J., Meyer A., Ma L., Wang X., Ming W. (2015). Terpolymer-based SIPN coating with excellent antifogging and frost-resisting properties. RSC Adv..

[23] Wang Y., Li T., Li S., Sun J. (2015). Antifogging and frost-resisting polyelectrolyte coatings capable of healing scratches and restoring transparency. Chem. Mater..

[24] Ezzat M., Huang C.J. (2016). Zwitterionic polymer brush coatings with excellent anti-fog and anti-frost properties. RSC Adv..

[25] Li Y., Fang X., Wang Y., Ma B., Sun J. (2016). Highly transparent and water-enabled healable antifogging and frost-resisting films based on poly(vinyl alcohol)-nafion complexes. Chem. Mater..

[26] Hong J.W., Cheon H.K., Kim S.H., Hwang K.H., Kim H.K. (2017). Synthesis and characterization of UV curable urethane acrylate oligomers containing ammonium salts for anti-fog coatings. Prog. Org. Coat..

[27] Yao B., Zhao H., Wang L., Liu Y., Zheng C., Li H., Sun C. (2018). Synthesis of acrylate-based UV/thermal dual-cure coatings for antifogging. J. Coat. Technol. Res..

[28] Bai S., Li X., Zhang R., Li C., Zhu K., Sun P., Zhao Y., Ren L., Yuan X. (2019). Enhancing antifogging/frost-resisting performances of amphiphilic coatings via cationic, zwitterionic or anionic polyelectrolytes. Chem. Eng. J..

[29] Zhang T., Fang L., Lin N., Wang J., Wang Y., Wu T., Song P. (2019). Highly transparent, healable, and durable anti-fogging coating by combining hydrophilic pectin and tannic acid with poly(ethylene terephthalate). Green Chem..

[30] Zhao J., Lu P., Song L., Tian L., Ming W., Ren L. (2020). Highly efficient antifogging and frost-resisting acrylic coatings from one-step thermal curing. Colloids Surf. A.

[31] Zhong H., Liu X., Yu B., Zhou S. (2022). Fast UV-curable zwitter-wettable coatings with reliable antifogging/frost-resisting performances. Biomimetics.

[32] Zhang W., Zhu L., Ye H., Liu H., Li W. (2016). Modifying a waterborne polyacrylate coating with a silica sol for enhancing anti-fogging performance. RSC Adv..

[33] Kaya A.S.T., Cengiz U. (2019). Fabrication and application of superhydrophilic antifog surface by sol–gel method. Prog. Org. Coat..

[34] Chang C.C., Lin Z.M., Cheng L.P. (2019). Preparation of superhydrophilic nanosilica/polyacrylate hard coatings on plastic substrate for antifogging and frost-resistant applications. J. Appl. Polym. Sci..

[35] Shi J., Xu L., Qiu D. (2022). Effective antifogging coating from hydrophilic/hydrophobic polymer heteronetwork. Adv. Sci..

[36] Baney R.H., Itoh M., Sakakibara A., Suzukit T. (1995). Silsesquioxanes. Chem. Rev..

[37] Kaneko Y., Iyi N., Kurashima K., Matsumoto T., Fujita T., Kitamura K. (2004). Hexagonal-structured polysiloxane material prepared by sol–gel reaction of aminoalkyltrialkoxysilane without using surfactants. Chem. Mater..

[38] Kaneko Y., Iyi N., Matsumoto T., Kitamura K. (2005). Synthesis of rodlike polysiloxane with hexagonal phase by sol–gel reaction of organotrialkoxysilane monomer containing two amino groups. Polymer.

[39] Toyodome H., Kaneko Y., Shikinaka K., Iyi N. (2012). Preparation of carboxylate group-containing rod-like polysilsesquioxane with hexagonally stacked structure by sol–gel reaction of 2-cyanoethyltriethoxysilane. Polymer.

[40] Kaneko Y., Toyodome H., Mizumo T., Shikinaka K., Iyi N. (2014). Preparation of a sulfo-group-containing rod-like polysilsesquioxane with a hexagonally stacked structure and its proton conductivity. Chem. Eur. J..

[41] Harada A., Shikinaka K., Ohshita J., Kaneko Y. (2017). Preparation of a one-dimensional soluble polysilsesquioxane containing phosphonic acid side-chain groups and its thermal and proton-conduction properties. Polymer.

[42] Kaneko Y. (2018). Ionic silsesquioxanes: Preparation, structure control, characterization, and applications. Polymer.

[43] Kaneko Y., Shoiriki M., Mizumo T. (2012). Preparation of cage-like octa(3-aminopropyl)silsesquioxane trifluoromethanesulfonate in higher yield with a shorter reaction time. J. Mater. Chem..

[44] Imai K., Kaneko Y. (2017). Preparation of ammonium-functionalized polyhedral oligomeric silsesquioxanes with high proportions of cagelike decamer and their facile separation. Inorg. Chem..

[45] Liu J., Kaneko Y. (2018). Preparation of polyhedral oligomeric silsesquioxanes containing carboxyl side-chain groups and isolation of a cage-like octamer using clay mineral. Bull. Chem. Soc. Jpn..

[46] Kozuma T., Kaneko Y. (2019). Preparation of carboxyl-functionalized polyhedral oligomeric silsesquioxane by a structural transformation reaction from soluble rod-like polysilsesquioxane. J. Polym. Sci. Part A Polym. Chem..

[47] Ishii T., Enoki T., Mizumo T., Ohshita J., Kaneko Y. (2015). Preparation of imidazolium-type ionic liquids containing silsesquioxane frameworks and their thermal and ion-conductive properties. RSC Adv..

[48] Maeda D., Ishii T., Kaneko Y. (2018). Effect of lengths of substituents in imidazolium groups on the preparation of imidazolium-salt-type ionic liquids containing polyhedral oligomeric silsesquioxane structures. Bull. Chem. Soc. Jpn..

[49] Kaneko Y. (2022). Superacid-catalyzed preparation of ionic polyhedral oligomeric silsesquioxanes and their properties, polymerization, and hybridization. J. Sol–Gel Sci. Technol..

[50] Maeda T., Hamada T., Tsukada S., Katsura D., Okada K., Ohshita J. (2021). Antifogging hybrid materials based on amino-functionalized polysilsesquioxanes. ACS Appl. Polym. Mater..

[51] Hamada T., Sugimoto T., Maeda T., Katsura D., Mineoi S., Ohshita J. (2022). Robust and transparent antifogging polysilsesquioxane film containing a hydroxy group. Langmuir.

[52] Kozuma T., Mihata A., Kaneko Y. (2021). Preparation of soluble poss-linking polyamide and its application in antifogging films. Materials.

[53] Nakagawa J., Morinaga S., Kaneko Y. (2024). Preparation of antifog hard coatings based on carboxy-functionalized polyhedral oligomeric silsesquioxane cross-linked with oligo(ethylene glycol)s. ACS Omega.

[54] Nardeli J.V., Fugivara C.S., Taryba M., Montemor M.F., Benedetti A.V. (2020). Self-healing ability based on hydrogen bonds in organic coatings for corrosion protection of AA1200. Corros. Sci..

[55] Liu T., Zhao H., Zhang D., Lou Y., Huang L., Ma L., Hao X., Dong L., Rosei F., Lau W.M. (2021). Ultrafast and high-efficient self-healing epoxy coatings with active multiple hydrogen bonds for corrosion protection. Corros. Sci..

[56] Bai Z.G., Bai Y.Y., Zhang G.P., Wang S.Q., Zhang B. (2021). A Hydrogen bond based self-healing superhydrophobic octadecyltriethoxysilane−lignocellulose/silica coating. Prog. Org. Coat..

[57] Mozhdehi D., Ayala S., Cromwell O.R., Guan Z. (2014). Self-healing multiphase polymers via dynamic metal–ligand interactions. J. Am. Chem. Soc..

[58] Yang S.M., Zhou S., Yuan J.Y. (2025). Self-healing elastomers and coatings via metal coordination bonds. Chem. Eur. J..

[59] Sinawang G., Osaki M., Takashima Y., Yamaguchi H., Harada A. (2020). Supramolecular self-healing materials from non-covalent cross-linking host–guest interactions. Chem. Commun..

[60] Lu P., Xu J., Tian W., Zhang C., Niu S., Zhao J., Ming W., Ren L. (2023). Robust antifogging coatings with ultra-fast self-healing performances through host–guest strategy. Chem. Eng. J..

[61] Liu Y.L., Chuo T.W. (2013). Self-healing polymers based on thermally reversible diels–alder chemistry. Polym. Chem..

[62] Chao A., Negulescu I., Zhang D. (2016). Dynamic covalent polymer networks based on degenerative imine bond exchange: Tuning the malleability and self-healing properties by solvent. Macromolecules.

[63] Chen K., Zhu H., Zhang Z., Shao Y., Yu Q., Cao X., Pan S., Mu X., Gao Z., Wang D. (2023). Self-healing polyurethane coatings based on dynamic chemical bond synergy under conditions of photothermal response. Chem. Eng. J..

[64] Wang H., Kuwayama M., Fujisawa Y., Oshima Y., Yamauchi Y., Wu J., Aida T. (2025). Poly(thioether thiourea)s as novel room-temperature self-healable glassy polymers. CCS Chem..

[65] Outwater J.O., Gerry D.J. (1969). On the fracture energy, rehealing velocity and refracture energy of cast epoxy resin. J. Adhes..

[66] Fang L., Chen J., Zou Y., Xu Z., Lu C. (2017). Thermally-induced self-healing behaviors and properties of four epoxy coatings with different network architectures. Polymers.

[67] Hornat C.C., Urban M.W. (2020). Entropy and interfacial energy driven self-healable polymers. Nat. Commun..

[68] England M.W., Urata C., Dunderdale G.J., Hozumi A. (2016). Anti-fogging/self-healing properties of clay-containing transparent nanocomposite thin films. ACS Appl. Mater. Interfaces.

[69] Wang S., Wang H., Fan Z., Yang H., Wang Q., Cai Z. (2021). Facile preparation of a high-transparency anti-fogging/frost-resisting poly(amps-*co*-aa) coating with self-healing property. Prog. Org. Coat..

[70] Yang Y., Zeng L., Li X., Cai Z. (2024). Hydrophilic/hydrophobic poly(AA-*co*-BA-*co*-BPA) anti-fog coating with excellent water resistance and self-healing properties. Prog. Org. Coat..

[71] Liang B., Zhong Z., Jia E., Zhang G., Su Z. (2019). Transparent and scratch-resistant antifogging coatings with rapid self-healing capability. ACS Appl. Mater. Interfaces.

