# Supplementary material for: Preparation of Self-Healing Antifogging Hard Coatings Using Carboxy-Functionalized Polysilsesquioxanes and Oligo(ethylene glycol)s
Source: Polymers (Basel). 2025 Sep 15;17(18):2491. doi: 10.3390/polym17182491 (PMC12473423; doi:10.3390/polym17182491)
Supplement: Supplementary file 1 [file polymers-17-02491-s001.zip › polymers-3834413-supplementary.pdf]

Supplementary Materials

Preparation of Self-Healing Antifogging Hard Coatings Using Carboxy-Functionalized Polysilsesquioxanes and Oligo(ethylene glycol)s

Seiya Morinaga [1], Rione Baba [1], Chino Fujii [2] and Yoshiro Kaneko [1,2,*]

[1] Graduate School of Science and Engineering, Kagoshima University, 1-21-40 Korimoto, Kagoshima 890-0065, Japan

[2] Faculty of Engineering, Kagoshima University, 1-21-40 Korimoto, Kagoshima 890-0065, Japan

* Correspondence: ykaneko@eng.kagoshima-u.ac.jp

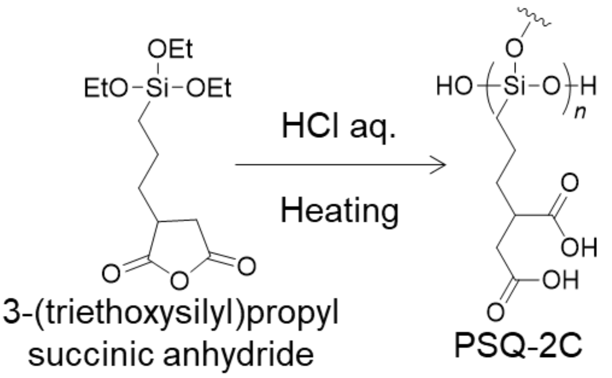

Scheme S1. Preparation of carboxy-functionalized polysilsesquioxane (PSQ-2C).

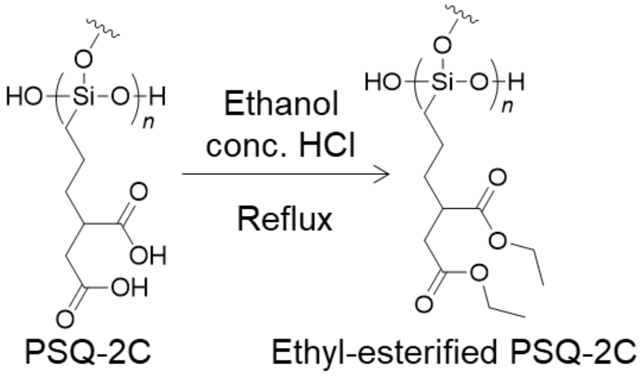

Scheme S2. Ethyl esterification of the carboxy groups in PSQ-2C.

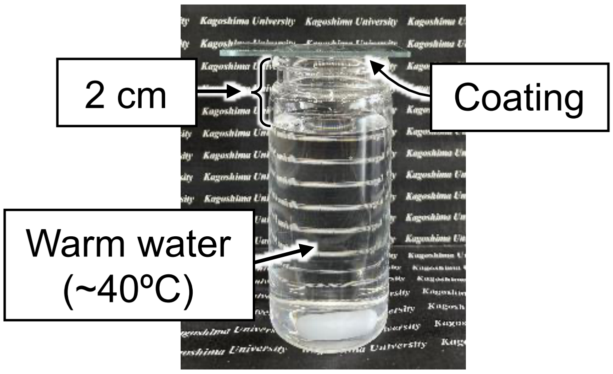

Figure S1. Photograph of the equipment used for antifogging evaluation.

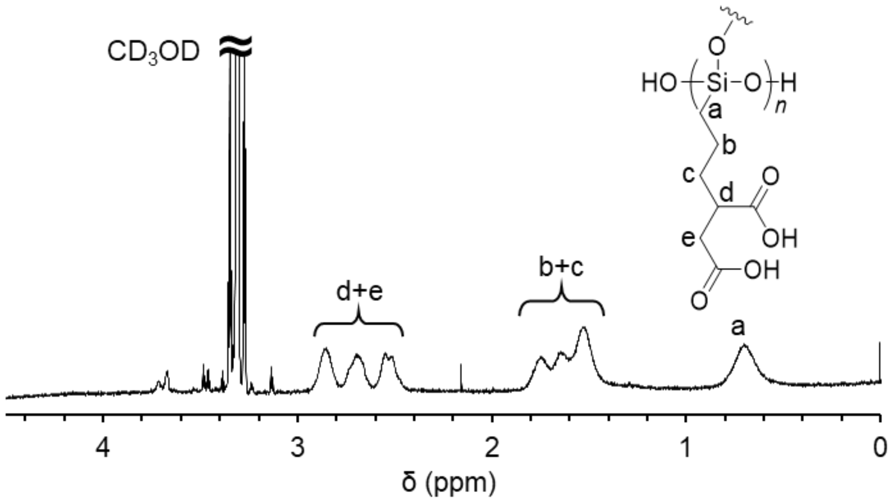

Figure S2. ^{1}H nuclear magnetic resonance (NMR) spectrum of PSQ-2C in methanol-d_4 (CD_3OD). Chemical shifts were referenced to CD_3OD (δ 3.31).

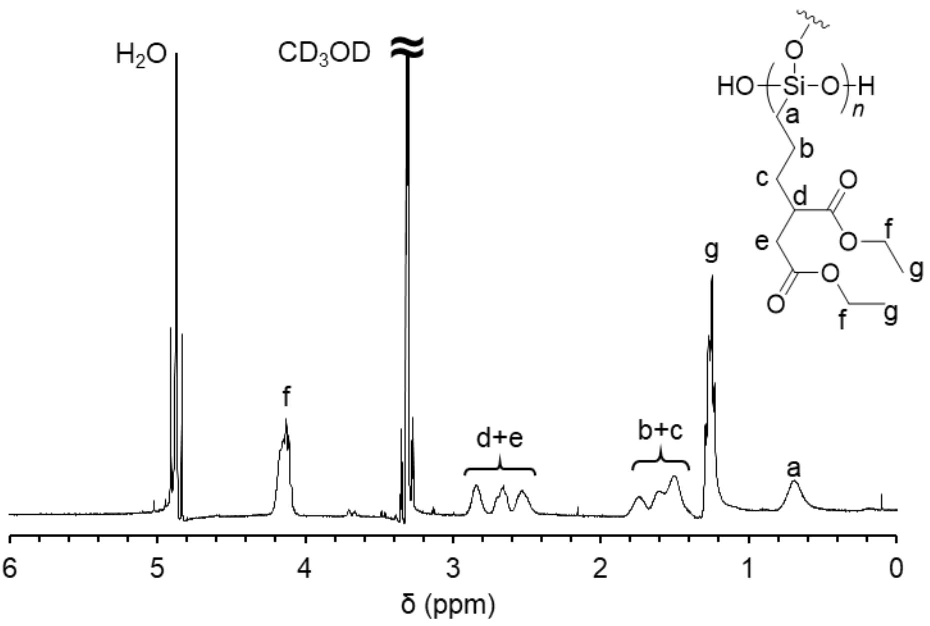

Figure S3. ^{1}H NMR spectrum of ethyl-esterified PSQ-2C in CD_3OD. Chemical shifts were referenced to CD_3OD (δ 3.31).

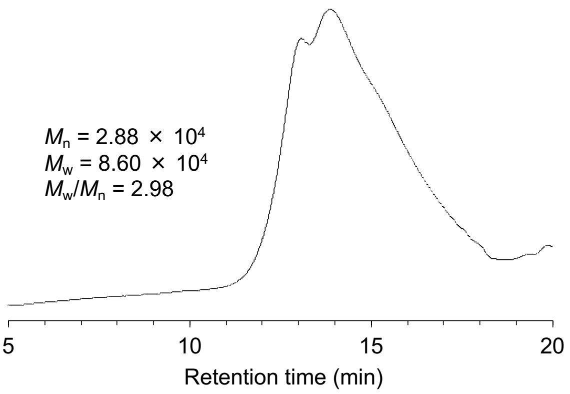

Figure S4. Gel permeation chromatography curve of ethyl-esterified PSQ-2C. Concentration: 0.5 w/v%; eluent: tetrahydrofuran; standard: polystyrene.

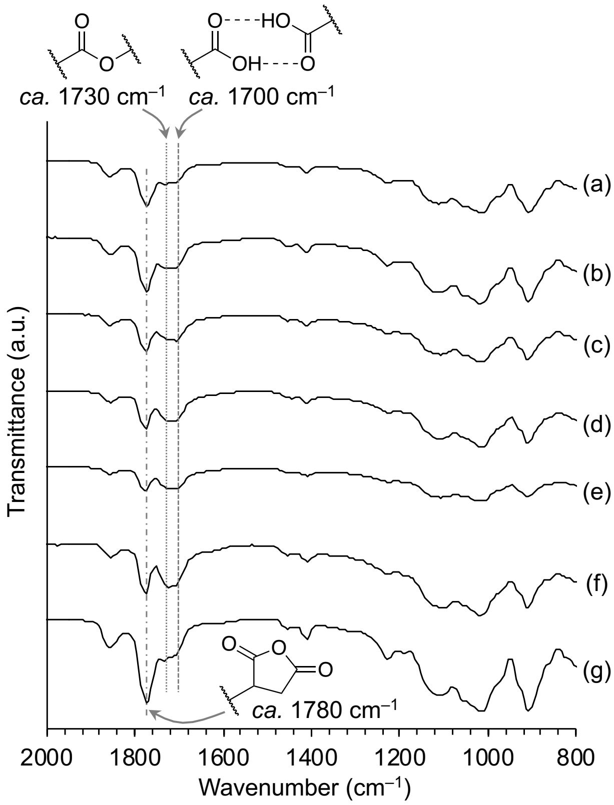

Figure S5. Fourier-transform infrared/attenuated total reflectance (FTIR/ATR) spectra of PSQ-2C/oligo(ethylene glycol) (OEG) ((a) $n = 1$, (b) $n = 2$, (c) $n = 3$, (d) $n = 4$, (e) $n = 5$, and (f) $n = 6$) coatings with a COOH/OH feed functional group ratio of 10:1 before water immersing for 1 h.

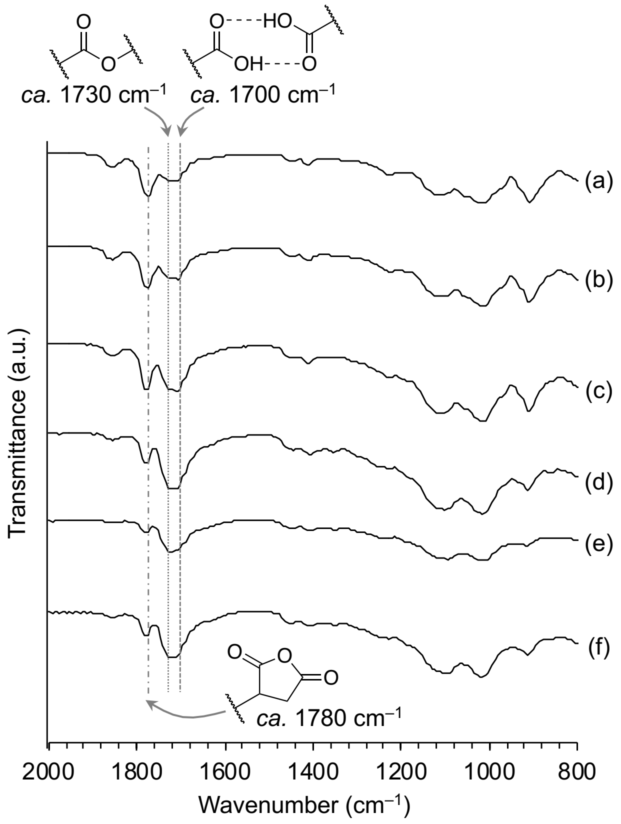

Figure S6. FTIR/ATR spectra of PSQ-2C/OEG ((a) *n* = 1, (b) *n* = 2, (c) *n* = 3, (d) *n* = 4, (e) *n* = 5, and (f) *n* = 6) coatings with a COOH/OH feed functional group ratio of 4:1 before water immersing for 1 h.

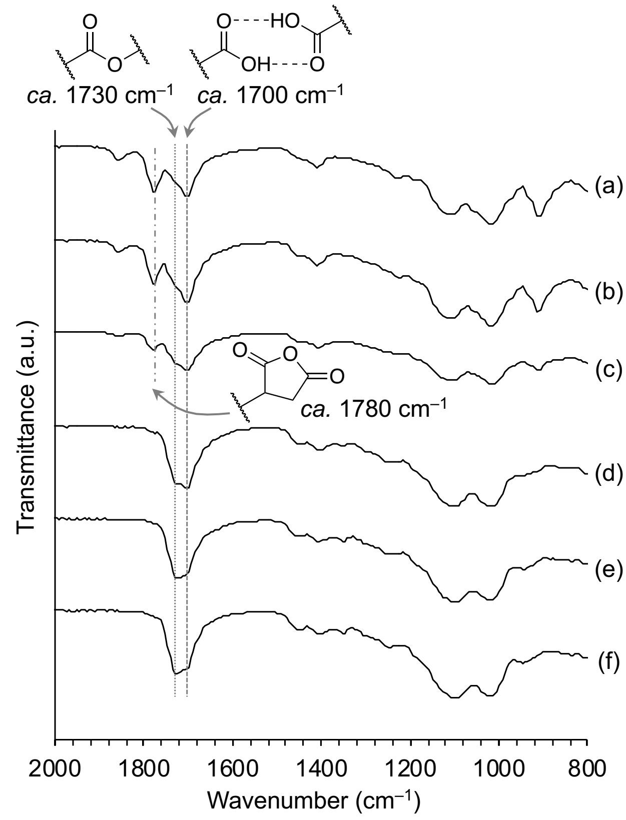

Figure S7. FTIR/ATR spectra of PSQ-2C/OEG ((a) n = 1, (b) n = 2, (c) n = 3, (d) n = 4, (e) n = 5, and (f) n = 6) coatings with a COOH/OH feed functional group ratio of 2:1 before water immersing for 1 h.

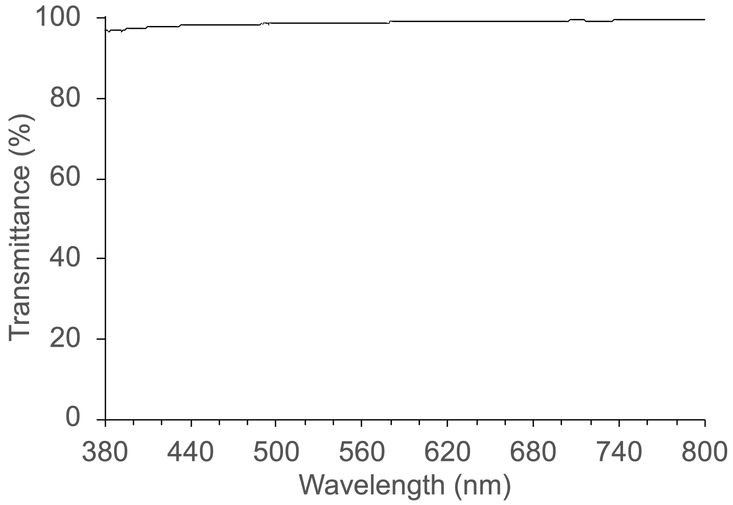

Figure S8. UV–Vis spectrum of the PSQ-2C/OEG (n = 4; COOH/OH = 10:1) coating.

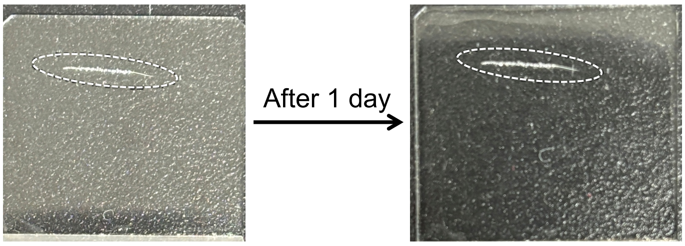

Figure S9. Appearance of the PSQ-2C/OEG (n = 4; COOH/OH = 10:1) coating after scratching with a cutter.
